# 

**Published:** 1897-03

**Authors:** 


CONTENTS.

| | PAGE |
|---|---|
| **The Effect of the Röntgen Rays on Calculi.** By JAMES SWAIN, M.S., M.D. Lond., F.R.C.S. Eng. ... ... | 1 |
| **The Symptoms and Treatment of Chronic Suppuration of the Maxillary Antrum.** By BARCLAY J. BARON, M.B. Edin. ... ... ... | 13 |
| **Secondary Rashes in Scarlet Fever.** By J. O. SYMES, M.D. Lond.... ... | 20 |
| **Three Cases of Vaginal Hysterectomy for Malignant Disease.** By W. H. C. NEWNHAM, M.A., M.B. Cantab., M.R.C.S. Eng. ... ... ... | 24 |
| **Two Cases of Compound Depressed Fracture of Frontal Bone.** By ARTHUR W. PRICHARD, M.R.C.S. Eng. ... ... ... ... ... ... ... | 27 |
| **A Case of Hysterical Disease of both Hips and both Knees, with Extreme Distortion of the Lower Limbs.** By CHARLES A. MORTON, F.R.C.S. Eng. ... ... ... | 30 |
| **Progress of the Medical Sciences:** | |
| **Medicine.** THEODORE FISHER, M.D.; J. MICHELL CLARKE, M.D. ... | 33 |
| **Surgery.** W. M. BARCLAY ... ... | 43 |
| **Ophthalmology.** F. R. CROSS, M.B. | 48 |
| **Reviews of Books** ... ... ... ... ... | 53 |
| **Notes on Preparations for the Sick** | 83 |
| **Meetings of Societies** ... ... ... ... .. | 87 |
| **The Library of the Bristol Medico-Chirurgical Society** ... ... ... ... | 96 |
| **Local Medical Notes** ... ... ... ... | 99 |
| **Scraps** ... ... ... ... ... ... ... ... | 101 |

